# Acute respiratory failure secondary to a cervical goitre in a pregnant woman: a case report

**DOI:** 10.1186/s12873-019-0231-8

**Published:** 2019-01-29

**Authors:** Sidi Driss El jaouhari, Nawfal Doghmi, Hamza Najout, Massine El hamouni, El hassane Kabiri, Hicham Bekkali, Jaafar Salim Lalaoui, Mustapha Bensghir

**Affiliations:** 1 Department of anesthesiology and critical care, Military Hospital Mohamed V, Faculty of medicine and pharmacy, university of Mohamed V Souissi, Rabat, Morocco; 2 Department of thoracic surgery, Military hospital Mohamed V, Faculty of medicine and pharmacy, university of Mohamed V Souissi, Rabat, Morocco

**Keywords:** Acute airway obstruction, Goitre, Pregnancy, Respiratory arrest

## Abstract

**Background:**

Pregnancy constitutes a significant factor in thyroid hypertrophy and can rarely progress to respiratory distress. We describe case of pregnant woman with acute respiratory distress following a tracheal compression due to goiter, quickly resulting in respiratory arrest, requiring emergency orotracheal intubation and thyroidectomy.

**Case presentation:**

A pregnant woman with a growing goiter was referred to the hospital with a respiratory difficulty. During the examination, we found a large homogeneous goiter. The patient showed signs of respiratory exhaustion with bradypnea and pulmonary auscultation revealing decreased ventilation of the two pulmonary fields. The evolution quick led to respiratory arrest. The patient was rapidly intubated, which saved her.

A thoracic computed tomography was performed and revealed a large goiter, compressing the trachea in its thoracic area and oppressing the vascular structures. Obstetrical ultrasound was normal. Thyroidectomy was decided after the patient’s preparation. After 24 h, the patient was successfully extubated without incident and the postoperative period was uneventful.

**Conclusion:**

Airway obstruction during pregnancy secondary to goiter is rare but can be fatal. Early diagnosis might have avoided the evolution towards the respiratory failure. Prevention requires early surgery preferably before pregnancy or in our case a surgery in the second trimester.

## Background

Pregnancy is known to be thyrogenic and may exacerbate features of thyroid disease [1]. Unusual increases in glandular size were observed, most often occurring in women who had previous goiter before pregnancy, who had not been diagnosed and therefore not been treated [2]. In some cases, term pregnant women underwent caesarean section followed by thyroidectomy to remove the hypertrophic goitre causing airway obstruction [3]. However, as the case described by Aloumanis and al., this compression may be the cause of acute respiratory failure, especially in the presence of a delayed etiological diagnosis, thus requiring an urgent thyroidectomy, [3].

We describe an unusual case of a pregnant woman, suffering from acute respiratory distress following a tracheal compression by goiter. This quickly led to respiratory arrest requiring emergency orotracheal intubation and thyroidectomy.

## Case presentation

A 31-year-old woman was referred to our emergency department with a shortness of breath. Mother of 3 children, she is in her 32nd week of pregnancy. At the beginning of the current pregnancy, swelling of the cervix appeared the size of which gradually increased, for which no consultation or diagnostic test was performed.

In fact, the symptoms began 7 days before admission. The patient had an increasingly aggravating dyspnea, first on exertion, then at rest, with notion of orthopnea, evolving into a context of apyrexia, which required an urgent consultation in our hospital.

On examination she was found to be severely dyspneic, an inspiratory stridor was audible, her respiratory rate was 40 breaths/min, her heart rate was 120 beats per minute, her blood pressure was normal (120–82 mmhg), and her Spo2 was 87% on room air. Inspection and cervical palpation revealed a large goiter. It was homogeneous without palpable nodules. A compression of the trachea by goiter was suspected. Given the patient’s gradual onset of dyspnea and anxiety, she was admitted to the observation room after oxygenation intranasally. Thoracic surgeons and gynecologists have been informed. Shortly after, the patient showed signs of respiratory struggle and her conscience began to deteriorate. During pulmonary auscultation, ventilation in both lungs was reduced. A diagnosis of acute respiratory obstruction secondary to enlarged goiter was very likely. An alert was triggered and she was immediately transferred to the intensive care unit (ICU).

In the ICU, after monitoring, an arterial gasometry was performed. The intubation equipment and induction drugs were ready. Arterial gasometry has revealed the following values (Pao2 = 58 mmHg; Paco2 = 81 mmHg; PH = 7, 09; Sao2 = 86%). The patient begins to show signs of respiratory exhaustion with bradypnea, progressing rapidly to respiratory arrest. The heart rate began to drop and blood pressure dropped to 79–35 mmhg. The decision was made to intubate the patient using rapid sequence induction. The patient was preoxygenated quickly without ventilation and induced. Etomidate was administered at a dose of 0.3 mg / kg followed by succinylcholine at the dose of 1.5 mg / kg. Sellick maneuver was not possible because of goiter compression. The intubation was attempted by the direct laryngoscopy and showed a central grade 1 view of the larynx (based on Cormack and Lehane classification). Intubation was performed using an intubation stylet and a lubricant gel. A size 6.5 cuffed polyvinyl chloride endotracheal armed tube (ETT) was passed after feeling a weak resistance. Correct positioning of the (ETT) was confirmed by pulmonary auscultation. After manual ventilation, respiratory distress was relieved and the hemodynamic state stabilized.

**Fig. 1:**
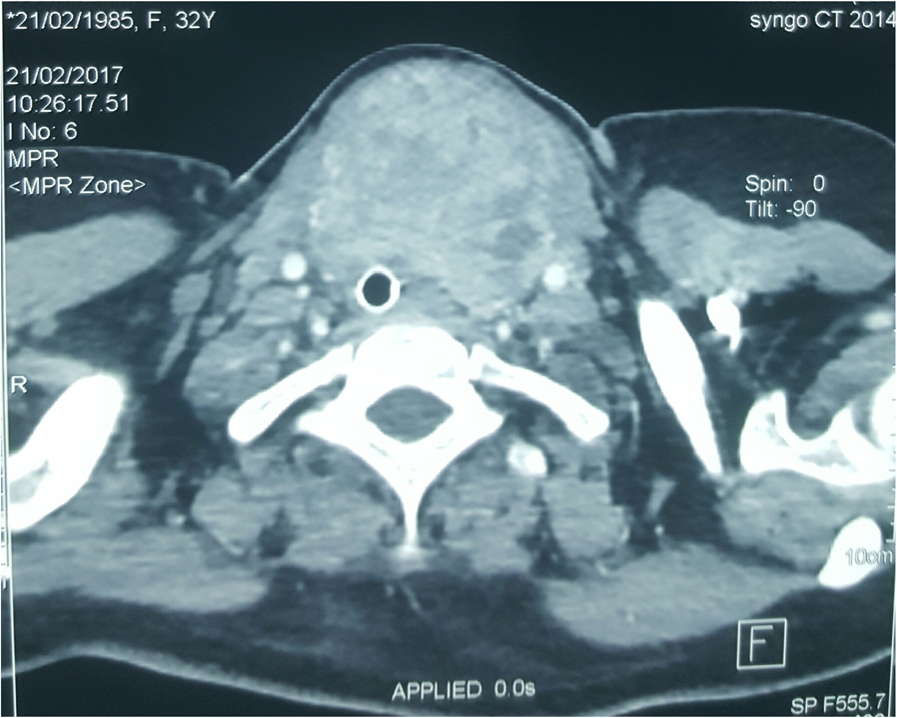
Computed tomography scan showing a huge goitre

A cerebral and thoracic computed tomography (CT) scan were performed and revealed a parenchymal nidus secondary to pneumonitis by inhalation and a large goiter plunging into the upper mediastinal orifice, 2 cm below the thoracic orifice, heterogeneous, measuring 71x44x100 mm, including an intrathoracic portion of the trachea and repressing the vascular structures which remain permeable (Fig. 1). The thyroid function tests were within normal range, with thyroid stimuling hormon (TSH) = 0,27μUI/ml, free triiodothyronine (FT3) = 5,6 pmol/l and free thyroxine (FT4) = 11 pmo/l. An obstetric ultrasound performed found a viable fetus and a positive cardiac activity. A thyroidectomiy was decided 48 h later, after preparation by corticotherapy (dexamethasone at 12 mg per day for two days), to accelerate fetal lung maturation.

**Fig. 2:**
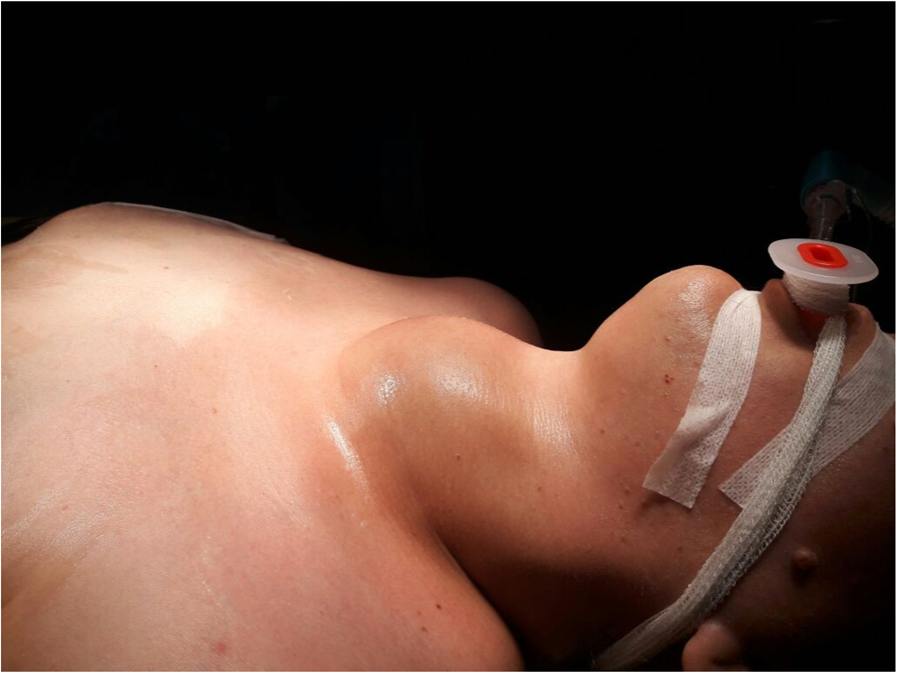
Lateral view of the patient’s neek, showing massive goitre

**Fig. 3:**
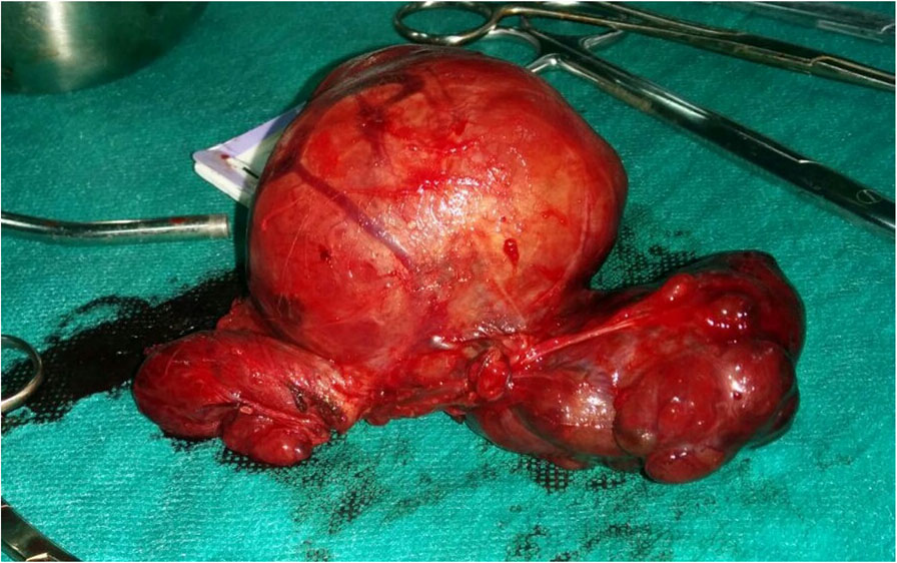
Excised thyroid gland

As expected, the patient was admitted into the operating room after 48 h (Fig. 2). The thyroid gland was found to extend in the intrathoracic part of trachea, causing severe concentric constriction of the airway. Vascular structures were slightly repressed. The removal was completed successfully by a combination of traction and blunt dissection. A total thyroidectomy was performed (Fig. 3). Tracheomalacia was suspected because of the dysmorphic shape of the intrathoracic trachea in combination with the slightly softer tracheal cartilage on palpation after goiter resection. No intraoperative tracheostomy was necessary and the surgery was completed. Prior to reversal of residual neuromuscular blockage and after careful aspiration of the oropharynx, the tube balloon was deflated. No leaks were observed, and the diagnosis of tracheomalacia was almost confirmed. It was decided to leave the endotracheal tube in situ for 24 h. She was transferred back to the ICU where an extubation was attempted the next day and ended without incident. After a stay in the thoracic surgery department, the patient was discharged from the hospital on the 5th postoperative day without any complications. The pathology showed multinodular goiter with areas of atypical hyperplasia without no sign of malignancy, it mesured 13x9x5,5 cm. Patient was treated with levothyroxin sodium tablets 75mcg/day. Four weeks later, the patient gave birth to a healthy new born with no significant abnormalities.

## Discussion

Pregnancy is known to be goitrogenic, with an increase of up to 30% in the size of the normal thyroid gland reported during pregnancy. This increase is mainly due to the HCG action; this hormone consists of two sub-units, one of wich is identical to TSH [1]. Adesunkanmi and Makinde reported an incidence of varying degrees of thyroid enlargement in 92.7% of pregnant women attending antenatal clinics in Ile-Ife teaching hospital in the south –west of Nigeria [4].

A Tracheal compression due to enlargement of the thyroid is quite common, rarely causing respiratory distress. Miller MR et al., have reported a prevalence of tracheal compression following 31% thyroid enlargement [3]. In pregnant women, this compression is rare. In addition, the coexistence of orthopnoea, caused by the reduction of lung volume as a result of diaphragmatic displacement upward, contributes to the severity of the respiratory distress [3]. Regarding our patient the rapid progression to respiratory arrest is related to the addition of several factors: obstructive respiratory failure due to compression by tracheal goiter, restrictive respiratory failure caused by decreased diaphragramitic mobility, the shunt effect related to the decrease of the pulmonary volume and in particular the maintenance of the late goiter before or during the first months of pregnancy as well as the underestimation of the respiratory prognosis in the hospital.

Surgery is always indicated for patients with severe airway obstruction in case of enlargement of the thyroid. Reviews concerning Timing of thyroid surgery during pregnancy recommend that postpartum surgery be postponed, unless there is evidence carcinoma or worsening respiratory complications [1]. In the third trimester, the thyroid surgery can cause labor. Reid and al performed cesarean section followed by thyroidectomy during term pregnancy [3]. In our patient, the clinical diagnosis of goiter was obvious, but for financial reasons, no biological or radiological evaluation was done before. The evolution was fatal to respiratory arrest following the compression of the trachea by huge goitre, hence the immediate intubation to save the patient. Thyroidectomy was performed 2 days later after pretreatment with corticosteroids.

Good management of the airway in case of acute respiratory distress, is essential to the patient’s survival. The existence of a goiter makes Sellick’s maneuver impossible as well as the tracheal approach and retrograde intubation in case of an emergency. Szeto and Hung [5] reviewed the mechanisms by which intrathyroidal bleeding occurs after haemorrhage in a cyst after intubation associated with the use of external laryngeal pressure and coughing during intubation. They noted that external pressure and increased venous pressure due to either Valsava manoeuvres or positive pressure ventilation could lead to intrathyroidal bleeding. Some authors have described the use of the Laryngeal Mask airway (LMA) containing 80% helium oxygen, and in extreme cases extracorporeal membrane oxygenation [6]. But in emergency, the full stomach associated with significant tracheal deformation militates against the use of laryngeal mask. In our patient, the induction was performed by a rapid sequence without cricoid pressure to avoid any additional risk.

Tracheomalacia is among the postoperative complications to be feared in case of prolonged tracheal compression. A simple and economical method of detecting tracheomalacia on the operating table at the end of surgery and before endotracheal extubation is described by Sinha et al. [1]. Before reversing residual neuromuscular blockade and after thorough suctioning of the oropharynx, the cuff of the tube is deflated. Tracheomalacia would cause the trachea to collapse over the tube, preventing peritubal leak after cuff deflation. The presence of a leak would exclude tracheal collapse. In our patient, intraoperative inspection of the trachea by the surgeon revealed malacic areas, tracheal cartilage seems less rigid on palpation after withdrawal of goiter and the diagnosis was confirmed by the method described by Sinha et al. we decided to keep the patient intubated for twenty-four hours of surveillance. An early extubation was attempted without incident. Fortunately, no complication of this type has occurred in our patient.

## Conclusion

Airway obstruction in pregnancy secondary to goiter is rare but can be fatal. Early diagnosis in our patient during her first consultation could have prevented the evolution towards a respiratory arrest. Prevention requires early surgery better before pregnancy or in our case, in the second trimester, to avoid endanger the life of the mother and the fetus.

## Abbreviations

CT: Computed tomography
ETT: Endotracheal tube
FT3: Free triiodothyronine
FT4: Free thyroxine
HCG: Human chorionic gonadotropine
ICU: Intensive care unit
Paco2: Arteriel pression in carbon dioxide
Pao2: Arteriel pression in oxigen
Spo2: Blood oxygen saturation
TSH: Thyroid stimuling hormone

## References

[1] Okeke CI, Merah NA, Atoyebi OA, Adesida A (2006). Acute airway obstruction in the puerperium secondary to massive thyroid enlargement. Int J Obstet Anesth.

[2] Glinoer D, Demeester R, Lemone M, Larsimont D, Andry G (2003). Acute increase in goiter size during a normal pregnancy: an exceptional case report. Thyroid.

[3] Aloumanis K, Mavroudis K, Vassiliou I, Arkadopoulos N, Smyrniotis V, Kontoyannis S (2006). Urgent thyroidectomy for acute airway obstruction caused by a goiter in a euthyroid pregnant woman. Thyroid.

[4] Adesunkanmi AR, Makinde ON (2003). Goitre prevalence in pregnant women attending antenatal clinic in a teaching hospital. J Obstet Gynaecol.

[5] Szeto LD, Hung CT (2002). Haemorrhage of a thyroid cyst as an unusual complication of intubation. Anaesth Intensive Care.

[6] Shao Y, Shen M, Ding Z, Liang Y, Zhang S (2009). Extracoporeal menbrane oxygenation assisted resection of goiter causing severe extrinsic airway compression. Ann Thorac Surg.

